# The Views of Syrian Immigrant Women on Family Planning and Unplanned Pregnancy: A Qualitative Study

**DOI:** 10.3389/ijph.2025.1607967

**Published:** 2025-02-20

**Authors:** Eda Yakıt Ak, Özden Tandoğan, Ergül Aslan

**Affiliations:** ^1^ Dicle University Atatürk Health Services Vocational School, Diyarbakır, Türkiye; ^2^ Istanbul Arel University Faculty of Health Sciences Department of Nursing, Istanbul, Türkiye; ^3^ Istanbul University-Cerrahpasa Florence Nightingale Faculty of Nursing Department of Women’s Health and Diseases Nursing, Istanbul, Türkiye

**Keywords:** migration, women, family planning, unplanned pregnancy, curettage

## Abstract

**Objectives:**

This study aimed to determine the views of Syrian immigrant women on family planning and unplanned pregnancies.

**Methods:**

The study was conducted using a phenomenological design, one of the qualitative research methods. The study data were collected using a semi-structured questionnaire, and an “inductive content analysis technique” was used to analyze the data.

**Results:**

The study identified four main themes. The first theme, “The Meaning of Having Children,” reveals that women view children as the essence of their lives and as a source of security for the future. The second theme, “Opinions on the Number of Children,” highlights that financial concerns and pressure from family and the surrounding environment play a significant role in decisions regarding the number of children. The third theme, “Views on Family Planning,” shows that while women are aware of family planning methods, their use is often limited due to economic, cultural, and religious factors. Lastly, the fourth theme, “Views on Unplanned Pregnancies,” indicates that unplanned pregnancies are commonly regarded as “God’s will,” and abortion is generally deemed inappropriate due to religious beliefs.

**Conclusion:**

Cultural, religious, and economic factors significantly affect women’s access to and use of family planning services.

## Introduction

Due to the ongoing internal conflicts in Syria, a significant number of women have become refugees, with 49% of them being of reproductive age [1]. Deteriorating living conditions due to the nature of migration have negatively affected women’s access to health services, especially reproductive health and family planning services [2–4]. Limited access to family planning has resulted in higher birth rates in countries where women migrate compared to their home countries [5, 6]. According to Turkey Demographic and Health Survey (TDHS) 2018 results, a Syrian migrant woman gives birth to 5.3 children on average [7].

Some Syrian refugee women are unable to make personal decisions and experience difficulties in accessing family planning services due to their husbands’ wishes, cultural pressures, and low social status [2]. This situation prevents migrant women from having the necessary information and accessing family planning solutions. However, it is known that in 2009, 88% of Syrian women received family planning counseling in their home countries, and 83.6% of them had their family planning requests met [8]. In a comprehensive study conducted in post-migration Turkey, it was reported that 24% of Syrian women used modern methods, and 35% had an unmet need for family planning [9]. The most important consequence of unmet needs for family planning is unintended pregnancies [10]. Family planning offers both health and social benefits to women. It saves lives by preventing unwanted, unintended, and unplanned pregnancies (which can often be unsafe and illegal), reducing the need for abortion, and reducing a woman’s likelihood of death from pregnancy and childbirth-related causes [11]. It is important to include Syrian migrant women in education and awareness-raising programs, facilitate their access to family planning services, and remove social and cultural barriers to these services [12]. A study conducted among women migrating from Syria to Turkey showed that factors such as lack of knowledge, cultural characteristics, religious beliefs, and economic status are effective in the use of family planning methods [13]. It has been revealed that Syrian migrant women have significant difficulties in accessing family planning services, but when these services are offered in line with women’s preferences, utilization of services can be increased [14].

Understanding Syrian migrant women’s views on family planning is important to identify their needs and contribute to the organization of health services to meet these needs. Research on this subject provides an opportunity to understand migrant women’s knowledge levels on family planning, intra-family communication, access to health services, and coping strategies for unplanned pregnancies. At the same time, determining views on family planning plays an important role in the formulation and implementation of health policies. Syrian migrant women’s views on family planning can contribute to the organization of health services to meet the needs in this area. In this regard, this study was planned to determine the views of Syrian immigrant women on family planning and unplanned pregnancies.

## Methods

This study was conducted between 16 March and 30 April 2024, using the phenomenology design, with face-to-face in-depth interviews.

The population of the study included Syrian women who applied to a non-governmental organization in Diyarbakır province. Social Assistance Rehabilitation and Adaptation Centre (SOHRAM - CASRA) in Diyarbakır province is a community-based non-governmental organization. The organization provides educational, social, and psychological support to Syrian migrants. Researcher EYA volunteers as a reproductive health counselor in the association.

The sample comprised 15 women who were contacted on the specified dates. The sample comprised women aged between 18 and 49 years who were married, had experience with pregnancy and childbirth, could communicate through an interpreter, and consented to participate in the study. Participants were determined by the “criterion-based sampling technique,” one of the purposive sampling methods.

Data were collected using a semi-structured questionnaire developed by the researchers based on the literature [14]. It included 15 questions about demographic characteristics, pregnancy, childbirth, and family planning methods. Migrant women were asked eight semi-structured open-ended questions about their thoughts on having children, family planning knowledge, unplanned pregnancies, and unplanned pregnancies. To assess the applicability of the semi-structured questionnaire, a preliminary interview was conducted with one woman. Following the interview, it was determined that no changes were necessary to the questionnaire. The participant form consists of two parts. The first section includes questions designed to gather demographic information, such as age, gender, marital status, education level, number of children, duration of residence in Turkey, and the use of family planning methods. The second part of the form consists of semi-structured questions. A pilot study was previously conducted to identify any issues with comprehension, using a semi-structured questionnaire comprising open-ended questions. Additionally, three pilot interviews were carried out with the assistance of an interpreter in an unused room at the association. Based on the findings, some of the prepared questions were rewritten to enhance clarity. The participants were not informed about the purpose of the study. There were no time constraints for the interviews. Interview durations varied between 25 and 30 min. All interviews were audio recorded and transcribed verbatim for later analysis.

Ethical approval was obtained from Dicle University Social and Human Sciences Ethics Committee (Date: 15.03.2024 Number: E-14679147-663.05-642434), and verbal consent was obtained from the women who voluntarily participated in the study. Written permission was obtained from the association where the study was conducted. The study was conducted following the principles of the Declaration of Helsinki.

Data were analyzed using the theoretical thematic analysis technique, which is widely used in qualitative research, and the analysis was handled in six stages [15]. (1) After each interview (within 24 h), the audio recordings from the in-depth interviews were carefully listened to and transcribed. Fifteen separate Word documents were produced, one for each participant. The printed documents were examined. Each document was reviewed several times, and the initial analytical notes were written alongside the statements. As a result, the goal of familiarity with the data was achieved. (2) The data items in all documents were coded. (3) Related codes were brought together, and preliminary themes were formed. (4) The comprehensibility of the themes that were created was evaluated. A descriptive analysis (code map) was performed using MAXQDA. (5) In the last stage, the researchers interpreted the participants’ perceptions of the subject through the themes that were created. To protect their privacy and the confidentiality of their identifying information, the participants were given codenames in the form of C1, C2, etc.

## Results

The mean age of the participants was 32.80 ± 3.745 (min-max: 24–40). All of the women are married, 40% of them are secondary school graduates, and 60% of them have an income lower than their expenses. The average duration of living in Turkey after migration is 9.33 ± 1.95 years (min-max: 7–12). All participants have children, and the average number of children is 3.80 ± 1.61 (min-max: 2–8). The socio-demographic characteristics of the women are presented in Table 1.

**TABLE 1:** Women’s socio-demographic characteristics (Diyarbakir, Turkey, 2024).

Case No	Age	Education status	Income status	Duration of residence in Turkey (years)	Number of children	Use of family planning	Method of family planning used
C1	30	Middle School	Middle	10	5	No	—
C2	32	Middle School	Low	8	6	Yes	IUD
C3	35	Middle School	Middle	10	4	Yes	Withdrawal
C4	36	Middle School	Middle	9	3	Yes	Oral contraceptive
C5	32	High School	Low	14	3	Yes	Oral contraceptive
C6	33	High School	Low	7	3	Yes	Condom
C7	29	High School	Middle	9	3	Yes	IUD
C8	33	Middle School	Low	10	4	No	—
C9	33	High School	Low	7	2	No	—
C10	40	Primary School	Low	7	8	No	—
C11	24	High School	Middle	9	3	Yes	Condom
C12	36	Primary School	Low	11	2	Yes	IUD
C13	33	University	Low	8	5	Yes	Withdrawal
C14	36	Middle School	Low	9	3	Yes	Oral contraceptive
C15	30	Middle School	Middle	12	3	Yes	IUD

The main themes and sub-themes determined by thematic analysis of the data obtained from the interviews are presented in Table 2.

**TABLE 2:** Main theme and subthemes (Diyarbakir, Turkey, 2024).

Main themes	Subthemes
1. The Meaning of Having Children	1.1. The meaning of life
	1.2. Reassurance of parents
2. Views on the Number of Children	2.1. Financial concerns
	2.2. Pressure from family
3. Views on Family Planning	3.1. Knowledge of family planning methods
	3.2. People who need to use family planning
4. Views on Unplanned Pregnancies	4.1. Religion-based perspective

### The Meaning of Having Children

Participants’ opinions on the meaning of having children in their lives were categorized under two headings (meaning of life, security of parents).

#### The Meaning of Life

Women stated that having children is the meaning of life and an important emotion that should take place in the flow of life. Some of the women’s statements defining children as the meaning of life are given below:

**C.9**…“*The child is necessary for me to bring good to his parents and to give his family a taste of life.”*

**C.10…** “*Children will take care of you when you are very old. Children are life for me. Life is empty without children. I have eight children.”*

#### Reassurance of Parents

When migrant women were asked about the importance and meaning of children for them, they stated that they consider children as the ones who will care for them in their old age. Some of the statements in which women defined children as security are given below;

**C.2…** “*A child gives confidence to his/her family, and if he/she is a good child, he/she can take care of his/her family in the future. The duty of parents is to raise good children. If you send children to school, they can get a good job and be a good son or daughter.”*

**C.11…** *“It is a good thing to have children; it is a good feeling. In my culture, as an Arab, they love and want boys more. They believe that a male child takes care of the family more… But for me, there is no such thing; I didn’t convince myself of that. Children are the only thing that will stay with me after I grow up.”*

### Views on the Number of Children

Two sub-themes were identified for the factors influencing the participants to have children (financial concerns, pressure from family).

#### Financial Concerns

Women stated that their low-income status was effective in having children. Some statements reflecting women’s financial concerns, which influence their decisions about having children, are provided below:

**C.9** *…* “*Both sides would say something about having a child. But now no one says anything because the financial situation is not good. My sister-in-law had five children, and she still wants to have more, but she is not in a financial situation. We always say don’t give birth anymore…”*

**C.13…** “*Two or three children are enough. We could have had many children before, but we were financially well off. Now our financial situation is bad, and we cannot take care of them. If there were more children, the house would be more cheerful, you would have a family, but we need money. When you bring a child into the world, you need to give it a good life. Good food, good clothes, good education… I mean, you can’t just bring it and throw it out on the street…”*

#### Pressure From Family

Women stated that they were under pressure, especially from their husbands’ families, regarding their marriages and having children. Some of the statements indicating spousal pressure are presented below:

**C.9…** *“When I first got married, I lived with my mother-in-law for three years. I didn’t know I was pregnant; I was doing housework, and I miscarried. I didn’t have children for two more years. My mother-in-law was always talking to my husband. My mother (mother-in-law) didn’t say anything to me, and she took me to the doctor to ask why the daughter-in-law couldn’t have children.”*

**C.11…** *“My mother and mother-in-law are harassing me because I am the only male child, there should be a sibling for that boy. I convinced my husband that since we cannot afford it, we can keep only one son. This is God’s work, after all. It cannot happen if he does not want it. My husband is convinced and now he doesn’t want it either.”*

### Views on Family Planning

Two sub-themes were identified regarding the participants’ knowledge, attitudes, and practices toward family planning (Knowledge of family planning methods and those who should use family planning).

#### Knowledge of Family Planning Methods

Women stated that they generally received information about family planning through family, close environment, and health institutions. Some statements regarding their knowledge of family planning methods are presented below:

**C.13…** *“Spiral, normal (withdrawal), injection, condom. As far as I heard from the hospital, health center, hospital… I used to ask when I went to the doctor, I heard it from the women around me, my brothers and sisters. I only used withdrawal. Yes, I mean, I have five children. I had one abortion of my own will. I had the children of my own will…”*

**C.11…** *“I got married when I was 16, but by force, I didn’t want to. Eight months later, I gave birth to a child. I didn’t know because I got married at a young age. At first, no one told me how to use protection. I learned about the spiral from my mother, and I heard about the medicine from my sisters-in-law.”*

#### People Who Need to Use Family Planning

Regarding those who should use family planning, women stated that those who have many children and those with poor income should especially use family planning. Some of the women’s statements about those who should use family planning are as follows:

**C.4…** *“For me, it is necessary because I don’t want too many children. Financially, childcare is difficult. When I give birth to four children, it is very important not only to give birth but also to take care of them. Financially poor women should wait and not have children right away.”*

**C.6…** *“Women who want to organize their families should use it… Families with child illnesses and women who are financially strapped should use it.”*

### Views on Unplanned Pregnancies

Participants’ views on their attitudes towards unplanned pregnancies were identified as two sub-themes (Religion-based perspective and condition of the child in the womb).

#### Religion-Based Perspective

All of the women stated that they found it right to leave themselves to the will of God and give birth to the child in the face of an unplanned pregnancy. Some of the women’s statements indicating their religious perspectives on unplanned pregnancies are presented below:

**C.2…** *“If she suddenly became pregnant, that child should not be touched because it is haram. It is haram, rabbilalemin… Allah has made it haram… If the child is in good health, if there is nothing wrong with the child, the child should be delivered. But if the child died in the womb, she can abort it.”*

**C.13…** *“According to our religion, if a month or two has passed, she has to accept it, it's haram, it’s a sin… But if 40 days have not passed, she can take medicine or something and abort the baby herself… I had an abortion once. I was very scared. Three months had passed, and I went to the doctor. And he said it was dead in my body; there was no pulse…”*

## Discussion

This study, which explored the views of Syrian migrant women on unplanned pregnancies and family planning, presents findings that could inform the more effective organization of health services.

The study revealed two different perspectives on the meaning of having children in women’s lives. On one hand, children are viewed as the essence of life and the future security of the parents. On the other hand, financial concerns and pressures from family and the surrounding environment also influence the decision to have children. For Syrian women, having children plays a central role in their lives and is seen as a future security. Having children gives meaning to their lives and strengthens their social status [2]. For Syrian women, having children reinforces their role within the family, demonstrates their fertility, and allows them to look to the future with hope. A child also assures in terms of providing people who will support and care for her in the future [13, 16]. In this study, similar to existing literature, women viewed their children as the meaning of their lives and an essential element that should be integrated into the flow of life [2, 13, 16]. Therefore, it can be said that having children is of great value to women and plays a very important role in terms of social status.

In this study, women were generally aware of family planning methods; however, their use was restricted by economic, cultural, and religious factors. Similarly, in the literature, in studies examining the barriers to the use of family planning methods by Syrian immigrant women, it was reported that they lacked knowledge on family planning, genital hygiene, and reproductive health, mostly could not benefit from family planning services and often used ineffective methods [12]. Another study emphasized that Syrian women’s fertility characteristics and family planning use are affected by social, economic, and cultural factors [17]. In addition, in the systematic review on women’s use of family planning methods and attitudes in Turkey, it was reported that 57.5% of women use family planning methods, but 17.4% do not have information about the method they use [18]. According to a report by the United Nations Population Fund, the demand for family planning among married women in Turkey increased significantly in the 5 years following 2013. The unmet need for family planning among Syrian women is particularly high, reaching 21%, which is twice the rate observed in Turkey overall. While the use of modern methods is low among Syrian women, the adoption of traditional methods is notably high. These data reveal that more effort and resources should be made in family planning service provision in Turkey [19].

This study reveals that economic, cultural, and religious factors play an important role in women’s access to and use of family planning methods. Similar to the literature, the impact of religious beliefs on the use of family planning methods by Syrian migrant women in Turkey has been examined. Religious beliefs and practices have been shown to impose significant restrictions on the use of contraceptive methods [20]. In a qualitative study examining the factors influencing modern contraceptive use among Syrian migrant women in Lebanon, religious and cultural norms were found to be significant barriers to the use of modern methods [21]. It is emphasized that religious and ideological factors play an important role in policy and service provision on family planning issues in countries in the Middle East [22]. In accordance with their religious beliefs, families may think that they can have as many children as God gives them with a fatalistic approach. The same approach is in question when there is an unplanned pregnancy. When a woman experiences an unwanted pregnancy, she thinks that it is a gift from God and that she should make it right. They think that unwanted pregnancies should only take place in cases that threaten the mother’s life.

There are many religious interpretations of the reasons why Islamic teachings oppose fertility restriction and family planning. While some interpretations consider the use of contraceptive methods as a sin, others state that having a child is a blessing from God and that it is inappropriate to interfere with it [23–25]. This can make access to family planning services difficult and prevent women from accessing these services. This study shows that religious and traditional barriers play an important role in Syrian women’s access to and use of family planning methods. It was stated that having a child is a blessing of God, and it is not appropriate to interfere with this. Especially in the case of pregnancy, they stated that they do not find abortion religiously appropriate. In the case of an unplanned pregnancy, women stated that they do not find it religiously appropriate to have an abortion if the baby is in good health and that they see it as “God’s will.” In addition, it was also found that women were under pressure from their families to have many children. This study contributes to women’s more effective access to family planning services and demonstrates that religious and cultural factors in this area should be taken into account.

Syrian women generally see unplanned pregnancies as “God’s will” and do not find abortion religiously appropriate. Similarly, it has been reported in the literature that Syrian immigrant women have a fatalistic approach to any pregnancy and that their religious and cultural approaches are oriented towards maintaining pregnancy in unplanned pregnancies [26–28]. In this context, it is crucial to develop responsive and effective policies that consider cultural norms and traditional beliefs. Such an approach can play a significant role in safeguarding and enhancing the reproductive health rights of migrant women.

In this study, Syrian migrant women stated that their poor economic situation constituted an important obstacle to family planning. Participants stated that they did not want to have many children due to financial constraints but still could not use family planning methods due to economic difficulties. Syrian migrant women expressed that while they would like to have more children, economic limitations prevent them from doing so. Additionally, they reported being unable to use family planning methods due to their limited financial resources. Studies have shown that economic hardship restricts Syrian women’s access to contraception, resulting in unwanted pregnancies. Furthermore, it is emphasized that economic barriers play a critical role in limiting Syrian migrant women’s access to safe abortion services [29, 30]. This may be similar for migrant women in Afghanistan, where recommendations have been made to address the issue of economic access [31]. Therefore, providing economic support for this group can play a critical role in protecting their reproductive health rights. In this context, economic support mechanisms need to be strengthened and expanded to increase Syrian refugee women’s access to family planning services and promote the use of modern contraceptives.

### Conclusion and Suggestions

The findings, organized under four main themes, reveal that cultural, religious, and economic factors significantly influence women’s access to and use of family planning services. Women see children as the meaning of their lives and parents’ security for the future. Financial concerns and family/environmental pressure are influential factors in having children. Women are knowledgeable about family planning methods, but their use is limited. Unplanned pregnancies are seen as “God’s will,” and abortion is not considered appropriate due to religious beliefs.

It is essential to conduct studies with a more homogeneous sample that explores the issue from various perspectives. To effectively organize health services, it is recommended to gain a deeper understanding of the needs and viewpoints of Syrian migrant women regarding unplanned pregnancies and family planning.

### Limitations

The limitations of this study arise from the fact that only 15 Syrian migrant women in Diyarbakır were interviewed, which restricts the generalizability of the findings. Future studies will address these limitations by including participants from various provinces and utilizing direct communication methods. In addition, by adopting a more comprehensive approach covering other issues such as health, education, and employment, the needs of Syrian migrant women will be better understood.
